# Acetabular revision using a total acetabular allograft

**DOI:** 10.4103/0019-5413.50860

**Published:** 2009

**Authors:** Rajesh Malhotra, Vijay Kumar

**Affiliations:** Department of Orthopaedics, All India Institute of Medical Sciences, New Delhi, India

**Keywords:** Acetabular revision, revision hip arthroplasty, total acetabular allograft, total hip replacement

## Abstract

The most challenging aspect of an acetabular revision is the management of severe bone loss, which compromises implant fixation and stability. We present a case of failed acetabular revision with extensive bone loss (Paprosky Type 3b) in a 50-year-old woman with rheumatoid arthritis, which was treated using total acetabular allograft. At a follow-up of 1 year and 3 months, the allograft had united with the host bone. This is the first report of the use of a total acetabular allograft for revision total hip arthroplasty in India. The total acetabular allograft allows the placement of the component closer to the normal hip center, provides initial stability for the acetabular component, and restores bone stock to the host pelvis.

Total hip replacement (THR) is a highly successful surgery to relieve the pain of an arthritic hip. The failure of total hip arthroplasty, however, often requires major reconstructive surgery. Bone loss often accompanies failed THR and can be the cause as well as the result of the failure. Septic or aseptic osteolysis, infection, wear, and instability are the major causes of bone loss associated with a failed THR.^1^–^4^ One of the most challenging aspects of an acetabular revision is the management of severe bone loss that compromises implant fixation and stability. The aims of an acetabular revision include the achievement of a stable bone coverage that can support the new acetabular component, restoration of the anatomy and bone stock for future revisions, and equalization of the leg length.

We present a case of failed acetabular revision with extensive bone loss that was treated using total acetabular allograft. This is the first report of the use of a total acetabular allograft for revision total hip arthroplasty in India.

## CASE REPORT

**Figure 1:**
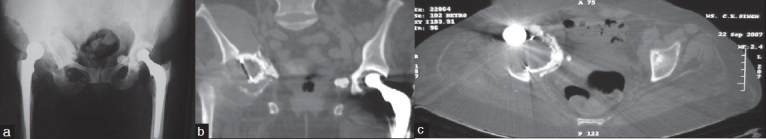
(a) Preoperative X-rays of the pelvis including both hips and thighs (anteroposterior view) of a 50-year-old woman with bilateral total hip replacement showing a type 3b Paprosky acetabular defect on the right side following revision total hip replacement done 2 months prior to presentation. Preoperative CT scan images, sagittal (b) and coronal (c) sections showing pelvic discontinuity and intra pelvic migration of the acetabular component

A 50-year-old woman underwent a bilateral total hip replacement for rheumatoid arthritis in 1999. Subsequently, a revision total hip replacement on the right side was done in May 2007 using a cemented acetabular component along with a bone substitute. She presented to us 2 months after this acetabular revision with complaints of severe pain in right hip and an inability to bear weight. She was bedridden and had a Harris Hip Score of 4. X-rays [Figure 1a] and CT scan [Figure 1b and c] revealed a failed acetabular component with a Type III b acetabular defect according to Paprosky classification.^2^ There was a pelvic discontinuity with superomedial migration of the acetabular component, break in Kohler's line, and complete loosening of the acetabulum at bone–cement interface.

Preoperative workup including bone scan, ESR, and CRP showed no evidence of any infection. The acetabular revision was done in accordance to the technique described by Paprosky *et al.*^2^

### Operative procedure

The hip joint was exposed using the posterior approach. The acetabular component was found to be lying loose. A thorough debridement revealed a large combined cavitary and structural defect along with pelvic discontinuity and loose pieces of bone mineral substitute lying in the defect area. After debridement and removal of the components, synovial white blood cell counts, gram staining of specimens of synovial fluid, and histological examination of frozen sections of inflammatory tissues were performed, and these excluded the possibility of infection.

All acetabular membranes were removed until the acetabular rim was completely exposed and bleeding bone encountered. An irradiated (25 kilogram) fresh frozen hemipelvis was procured from the bone bank. This allograft was thawed in povidine iodine and normal saline solution.

The acetabular allograft was shaped to buttress the host bone. The superior pubic and ischial rami of the graft were cut at a point distal to the acetabular confluence with a length remaining to fill the defects in the host pelvis. The iliac crest of the allograft was cut in a curvilinear manner from the greater sciatic notch to the anterior inferior iliac spine. Repeated fitting and trimming of the graft was performed to ensure the appropriate amount and placement of bone resection.

**Figure 2:**
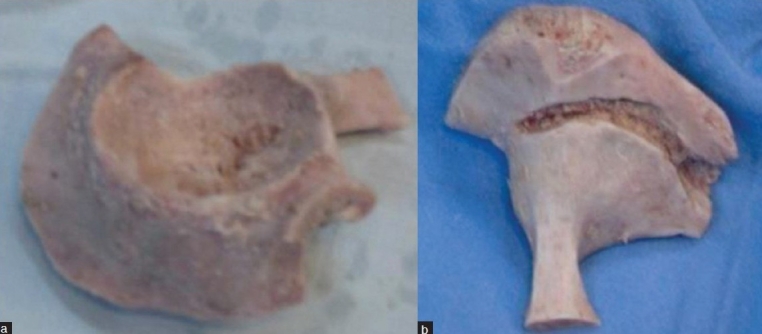
(a) Intraoperative photograph of the total acetabular allograft; lateral view and medial view. (b) The latter image shows the groove created with a burr

A tongue was created in the remaining rim of the host acetabulum. A groove was created in the medial aspect of the acetabular allograft using a burr [Figure 2a and b]. Such a tongue-in-groove mortise ensures an optimum contact and stability of the construct and also helps in union of the graft to the host. The acetabular allograft was held in a bone vice and reamed to remove the cartilage and minimum amount of subchondral bone. Morselized allograft was harvested from the remaining hemipelvis and packed into the defect.

The acetabular allograft was seated in place and manipulated to ensure that it lay in its most stable position. After further trimming the graft, it was impacted using an acetabular impactor to ensure a press fit in the tongue and groove mortise. Large-fragment, partially threaded cancellous-bone screws were placed in the superior rim of the iliac bone of the allograft to lag it to the thin iliac wing of the host allowing a bicortical purchase.

A Burch Schneider reconstruction cage (Zimmer, USA) was used to augment the fixation of the allograft. The acetabular allograft was then fashioned to ensure the support of cage along the posterior column. A cage with a diameter of 50 mm was chosen, as it allowed intimate contact with the host, and more importantly with the graft without over reaming and possible weakening of the bone.

The cage was then manipulated into position and fixed to the allograft bone and the remaining host bone using screws in the dome and iliac flange of the cage to ensure close approximation of the host bone and the allograft. An acetabular liner (size 47 mm) was then cemented into the cage. The morselized allograft was also impacted into the remaining defects and into the junction between the host and the allograft.

**Figure 3:**
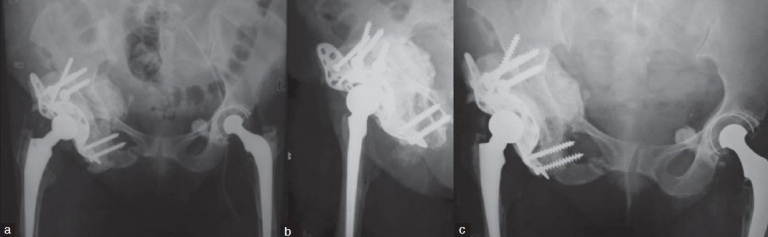
(a) Early postoperative X-rays of the pelvis including both hip and thighs (anteroposterior) and (b) lateral view of the right hip with thigh showing a well reconstructed acetabular defect using total acetabular allograft, morselized bone graft and Burch-Schneider reconstruction cage. (c) One-year follow-up X-rays of pelvis including both hips (anteroposterior view) showing graft union and incorporation

The femoral stem was not revised as it was found to be well fixed. A 28-mm femoral head with short neck length was implanted on the stem and a stable reduction was achieved [Figure 3a and b].

Postoperatively, the patient was kept from weight-bearing for 6 weeks. The patient was then mobilized toe-touch weight-bearing for another 6 weeks and progressive weight-bearing was started thereafter.

At a follow-up of 1 year and 3 months [Figure 3c], the allograft had united with the host bone and there is no evidence of any loosening, osteolysis, or resorption around the allograft. The patient is walking unaided and has a Harris Hip Score of 88.

## DISCUSSION

The treatment options of pelvic discontinuity and major acetabular defects in the setting of a revision total hip arthroplasty are use of a plate with cage and structural allograft, trabecular metal augments, custom-made implants, triflange implants, and whole acetabular allograft.^5^–^8^

The total acetabular allograft allows the placement of the component closer to the normal hip center. These grafts provide initial stability for the acetabular component and also restore bone stock to the host pelvis. These grafts unite with the host bone and provide a scaffold for future revisions.^5^,^6^

The use of a reconstruction cage improves the union of the graft to the host bone, as it dissipates the forces through the remaining pelvis, thereby preventing excessive transmission of force through the avascular graft. However, the use of acetabular reconstruction ring in isolation cannot be used to span a structural defect, as the cage will have no columnar support and repetitive loading would cause the cage to flex and lead to metal fatigue or pull out of screws. A structural acetabular allograft is required in conjunction with this cage to provide biological support to the cage and prevent fatigue fractures of the cage over the long term.^5^

It has been shown that the acetabular allografts unite at an average of 11 months (range, 6-16 months).^14^ Paprosky^6^ reported a success rate of 82% in cases where a cage was used along with total acetabular allograft as compared with 38% in cases without the use of cage. Garbuz^15^ also advocated the use of cage along with acetabular allograft for better results. A survival of as long as 20 years with an excellent functional outcome with radiographic evidence of graft incorporation and no signs of loosening has been shown by Gul *et al*.^12^ The acetabular allograft showed evidence of union at a follow-up of 15 months in our case; however, a longer follow-up is needed to evaluate the tendency of some of these massive allografts to resorb in the long term. [Table 1] provides the summary of the results of various reports on acetabular reconstructions using acetabular allografts.

**Table 1:** Summary of various reports on the use of acetabular allografts

Authors	Mode of reconstruction	No. of patients	Follow-up	Results	Complications
Macdonald^9^	Whole acetabular allograft and cemented cup without cage	17pts	31 months	13 grafts successful	Infection-4
					Migration-1
					Dislocation-6
Steihl^10^	Pelvic graft with plate and stabilization of anterior column	12	14-84 months	8 grafts successful	Infection-2
					Loosening-2
Schelfaut^11^	Deep frozen periacetabular graft with cemented cup without cage	14	42 months	Good results in 9	Mean resorption of 17% in 6 patients, 4 patients revised
Gul^12^	Acetabular allograft with cementless cup without cage	1	20 years	Excellent results	None
Paprosky^6^	Acetabular allograft and cemented cup without a cage	16	10 years	No loosening in 6 hips	6 hips revised due to loosening at 2.9 years
					Radiographic loosening seen in 4
Paprosky^6^	Acetabular allograft and cemented cup with a cage	48	2-8 years	20 cups had no loosening	9 hips revised for aseptic loosening, 9 cups had radiographic loosening
Saleh^13^	Pelvic allograft with cemented cup and cage	9	10.5 years	77% satisfactory results	3 revisions;1 for graft resorption, 2 for recurrent dislocation
Piriou^14^	Hemipelvic transplant with cemented cup without cage	20	4-10 years	65% good results	7 failures (5 aseptic loosening and 2 deep infections). Two dislocations
Garbuz^15^	Acetabular allograft with cage with cemented cup	8	5-11 years	7 (88%) successful	One failed due to infection
	Acetabular allograft with cemented cup without cage	14	5-11 years	12	2 failed
	Acetabular allograft with cemented cup	7	5-11 years	4	3 failed

A higher incidence of dislocation has been reported in these cases because of the difficulty in achieving a proper acetabular orientation. Although some acetabular components fail because of loosening, the restoration of bone stock by an acetabular allograft makes a revision operation much easier.

In spite of the concerns of graft resorption, collapse, and the transmission of infection,^16^ the use of acetabular allograft in acetabular revision surgery is useful to restore bone stock especially when the expectations are low.^17^

Patients with a Type 3B Paprosky acetabular bone defects were previously considered to be unreconstructable and were subjected to salvage procedures like girdlestone arthroplasty.^5^ A total acetabular allograft provides a viable alternative to treat these formidably challenging reconstructions. The availability of newer materials such as trabecular metal may facilitate the reconstruction in such cases minimizing the use of allografts.^6^ However, this option is at present neither easily affordable nor available in India.
